# New Treatment of Cancer*L’Experience, l. c.; and Lond. Lancet, April 11, 1840, p. 112.

**Published:** 1840-07

**Authors:** 


*New Treatment of Cancer.**—M. Jobert has endeavored to check the progress of this terrible malady, by tying all the vessels and dividing all the nerves which are distributed to the affected part. His efforts, however, have not been crowned with success.

In four cases of cancer of the lip M. Jobert tied the facial and coronary vessels, and divided the branches of the fifth nerve, which pass to the lip. The ligature of the vessels caused some improvement in the appearance of the ulcers, and on dividing the nerves the pain was removed; but in all cases he was compelled to extirpate the disease, at last.—*Ib.*

---

* L'Experience, l. c.; and Lond. Lancet, April 11, 1840, p. 112.

